# Evaluation of Surface Roughness of Translucent Zirconia After Applying Nd:YAG and CO2 Lasers: An In Vitro Study

**DOI:** 10.7759/cureus.64009

**Published:** 2024-07-07

**Authors:** Ahmad Shalabi, Chaza Kanout, Omar Hamadah

**Affiliations:** 1 Fixed Prosthodontics, Damascus University, Damascus, SYR; 2 Oral Medicine, Damascus University, Damascus, SYR

**Keywords:** continuous wave, co2 laser, nd:yag, cube x2, surface roughness

## Abstract

Background and objectives: Surface roughness is one of the most important factors that play an important role in increasing the connection between the surface of the tooth and the applied restoration. Due to the increased interest in zirconia and the improvement of its mechanical and aesthetic properties, studies have increased that work to improve and increase its surface roughness so that it can be used as a veneer in the future. This study aims to compare the effect of two types of lasers on the surface of highly transparent zirconia to evaluate the surface roughness resulting from the two techniques.

Methodology: The study sample consisted of 20 ceramic cubes made of translucent zirconia (DD cubeX2, Dental Direct, Germany). It was made using a CAD-CAM Zircodenta device (Imes-Icore, Germany) and a zirconia sintering furnace (Imes-Icore, Germany). The study sample was divided into two groups; the first group consisted of 10 cubes exposed to Nd:YAG laser and the second group consisted of 10 discs exposed to continuous wave CO_2_ laser. The surface roughness test was conducted for the study samples in each of the groups using a surface roughness tester. Data were collected and analyzed using SPSS v25 software.

Results: The surface roughness was measured and its mean was 1.208±0.22 in the Nd:YAG laser group and 0.809±0.21 in the CO_2_ laser group. There was a significant difference between the study groups according to the independent sample T-test.

Conclusion: This study concluded that the Nd:YAG laser surface roughens of zirconia is greater than the continuous wave CO_2_ laser, with a substantially significant difference.

## Introduction

The development of zirconia, its structure, and manufacturing methods have directed attention toward the use of highly transparent zirconia in the manufacture of veneers [1]. The current trend is to develop methods to ensure the bond of translucent zirconia to dental tissues by finding techniques to treat the inner surface of crowns and veneers made of translucent zirconia and increase the surface roughness, thus ensuring a clinically acceptable bond strength [2].

Zirconia is one of the types of polycrystalline dental ceramics. It has been widely used in dentistry due to the increasing demand of patients for metal-free restorations, as its optical properties are similar to those of natural teeth. Zirconia also has bio-receptivity and high mechanical properties, which is what made us use it in the manufacture of single crowns and fixed prosthodontics [3].

Translucency is one of the most important factors that affect the color match between natural teeth and restorations [1]. Translucency is the amount of light that passes through the restorative materials. The passage of light through the zirconia structure is affected by several factors (grain size, material density, and crystal structure) and to improve the aesthetic properties of zirconia restorations, the composition of traditional zirconia was modified by changing the percentage of impurities and the stabilizer. Those changes affected the structure of the crystals and thus the mechanical and aesthetic properties of the material, and translucent zirconia was produced, which was an alternative to E.max [4].

The main problem with translucent zirconia is its weak bond with dental tissues and its surface not being affected by the hydrofluoric acid used in treating E.max [5]. Therefore, research has turned to strengthening its adhesion to the enamel and dentin in various ways, including treating the surfaces of translucent zirconia before affixing them with various methods such as sandblasting and laser [6,7].

The laser is one of the recent methods used in dentistry, which has been used in treating soft and hard tissues [8]. Therefore, it has also been used in treating and roughening the surfaces of materials used in dental prosthetics [9].

Therefore, it was necessary to conduct a study to evaluate the surface roughness of highly transparent zirconia after laser treatment. This study aims to compare the effect of two types of lasers on the surface of highly transparent zirconia to evaluate the surface roughness resulting from the two techniques.

## Materials and methods

Study design

An experimental in vitro study was conducted to evaluate several lasers in surface roughness of highly translucent zirconia to study surface roughness.

Sample size estimating

The size of the studied sample was estimated using the Gpower software, based on preliminary data from the current study, where the results of this study were entered into the program, and using the Student's T test for independent samples, at a confidence of 0.95, and the effect size was 1.762. The level of significance is 0.05, and it turns out that the minimum sample size required is 8 in each group and raised to 10 to increase statistical safety.

Study sample

The study sample consisted of 20 ceramic cubes made of translucent zirconia (DD cubeX2, Dental Direct, Germany). It was made using a CAD-CAM Zircodenta device (Imes-Icore, Germany) and a zirconia sintering furnace (Imes-Icore, Germany). The study sample was divided into two groups; the first group consisted of 10 cubes exposed to Nd:YAG laser (1064 nm، 2.5 w، 20 Hz، 125 mj) at a distance of 1 mm from the surface of the cube for 45 seconds. The second group consists of 10 discs exposed to continuous wave CO_2_ laser (10600 nm، 4 w، 1000 Hz، 150 mj), at a distance of 1 mm from the cube surface for 1 minute.

**Figure 1:**
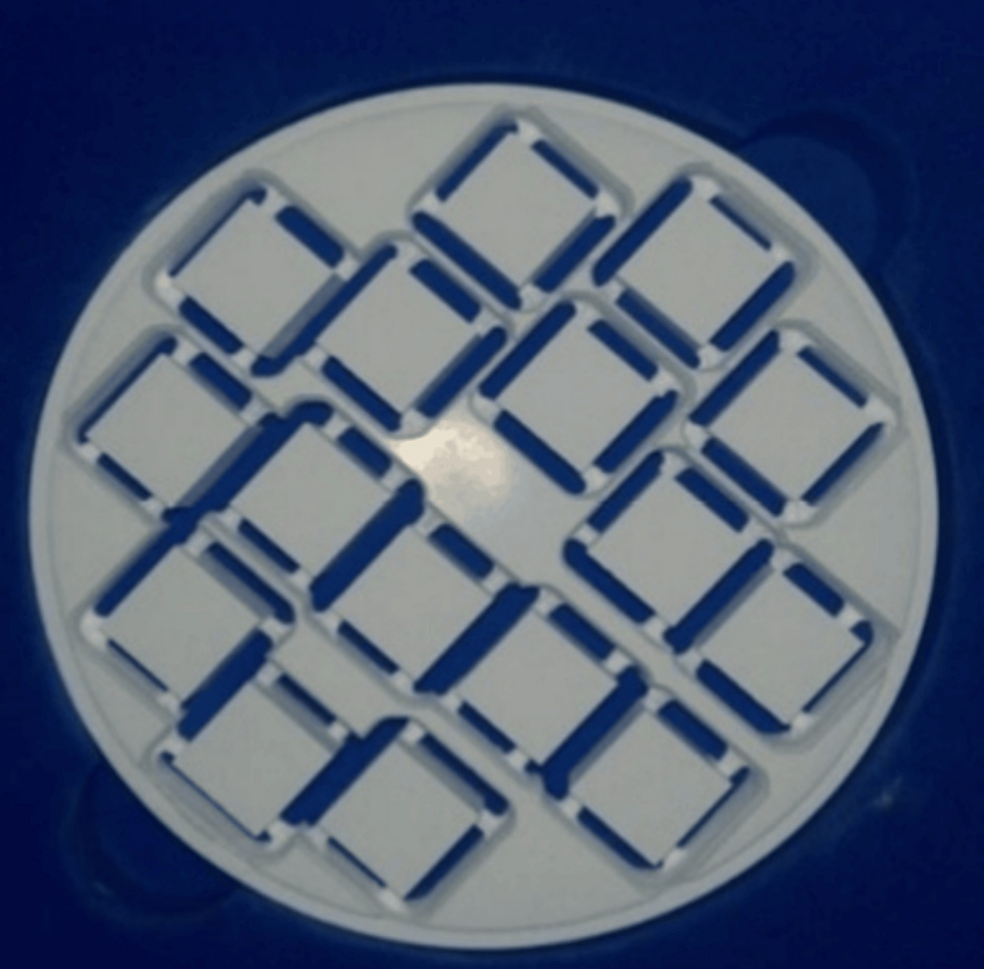
Translucent zirconia cubes after turning process.

The translucent zirconia blocks (DD cubeX2, Dental Direct, Germany) were cut into 20 cubes, each with dimensions of 10x10x10 (Figure 1) and then the samples were cleaned by placing them in an ultrasonic device with distilled water for 10 minutes.

The roughness of cubic zirconia was measured after turning and before the application of lasers using a surface roughness tester (TR200, TIME, USA). This is in order to determine whether turning cubic zirconia leads to an increase in its roughness. None of the study samples needed to be polished or smoothed before being treated with the laser because the turning did not affect the roughness of the zirconia cube.

The first group of cubes was treated using Nd:YAG laser (1064 nm، 2.5 w، 20 Hz، 125 mj) at a distance of 1 mm from the surface of the cube for 45 seconds, whereas the second group was treated using continuous wave CO_2_ laser (10600 nm، 4 w، 1000 Hz، 150 mj) at a distance of 1 mm from the cube surface for 1 minute.

Surface roughness measurement

The surface roughness test was conducted for the study samples in each of the groups using a surface roughness tester (TR200, TIME, USA). The sample was fixed on the base designated for measurement so that the measuring head was perpendicular to the surface of the cube, and then the measurement was carried out for each surface through mechanical contact between the sensor with the sample surface of 5 mm long for each measurement and at a speed of 0.5 mm per minute. To give Ra on its screen (roughness average), which represents the average roughness of each surface, the device electronically measures the mean of all the peaks and pits present over the entire measured distance of the surface.

Statistical analysis

Data were collected and entered into Excel and statistically analyzed using SPSS version 26 (IBM, SPSS Inc, USA), and an independent sample T-test was used to determine if there were statistically significant differences.

## Results

The sample of the current study was distributed into two groups (10 cubes in each group, 50%). Descriptive statistical analyses were conducted for surface roughness measurements of zircon cubes. The mean in the Nd:YAG laser group was 1.208, with a standard deviation of ±0.22, a minimum value of 0.682, and a maximum value of 1.425 (Table 1).

**Table 1:** Mean results of surface roughness.

Group	Number	Mean±SD	Min value	Max value
Nd:YAG laser	10	1.208±0.22	0.682	1.425
CO_2_ laser	10	0.809±0.21	0.452	1.081

The mean surface roughness in the CO_2_ laser group was 0.809, with a standard deviation of ±0.21, a minimum value of 0.452, and a maximum value of 1.081 (Table 1).

**Figure 2:**
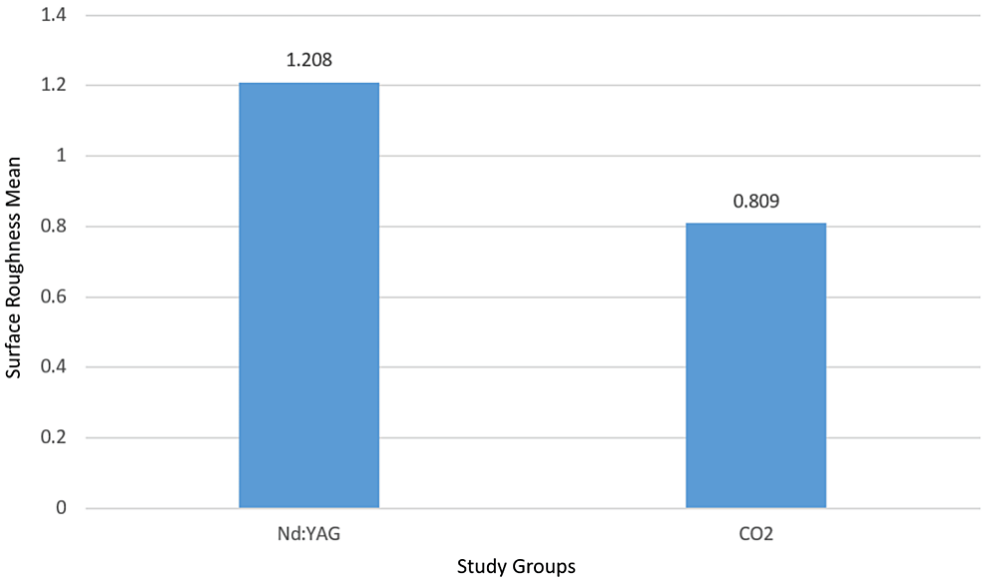
The mean values of surface roughness in both groups.

Figure 2 shows a comparison between the mean surface roughness of the Nd:YAG laser group and the CO_2_ laser group. 

We find from the previous values ​​that the average surface roughness value was higher in the Nd:YAG laser group. To find out if this difference is statistically significant, we test the data distribution with Kolmogorov-Smirnov test.

After conducting a test to determine the distribution of the data, it appeared that the P-value of the Kolmogorov-Smirnov test was greater than 0.005, and therefore the distribution of the data was normal (Table 2). Then the independent sample T-test was conducted to determine whether there are significant differences between the two study groups.

**Table 2:** Kolmogorov-Smirnov test to determine the distribution of the study sample. P-value <0.005 is a significant difference.

Measurement	Test value	Degrees of freedom	P-value
Surface roughness	0.185	20	0.200

The presence of differences between the two study groups was studied using the independent sample T-test, and the P-value was 0.001, and therefore, there is a significant difference between the two study groups.

Since the value of the independent sample T-test was higher in the Nd:YAG laser group, it outperforms the CO_2_ group according to Table 3.

**Table 3:** Independent sample T-test results for surface roughness. P-value <0.005 is a significant difference.

Group	Number	Independent sample T-test	P-value
Nd:YAG laser	10	12.903	0.001
CO_2_ laser	10	7.449	

From Table 1 and Table 3, we found that the surface roughness was greater in the Nd:YAG laser group than the CO_2_ laser group, with a statistically significant difference.

## Discussion

The use of zirconia has increased in various fields of dentistry in general, especially fixed prosthetics, as a result of the mechanical properties that allow it to withstand the pressures applied to the prostheses in the posterior areas, high resistance to wear, good color stability, and low thermal conductivity [10].

Improving the stability of zirconia is important to avoid loosening of the prosthesis, which is considered the third reason for replacing the prosthesis within a period of three years and the second reason for the failure of zirconia restorations after the fracture of the covering porcelain [11].

In 1981, research in the field of lasers was directed toward developing modern technologies that replace rotary drilling tools, and this led to the creation of the first Er:YAG laser, mediated by Keller and Hibst, that enables the cutting of hard dental tissues [12].

The results of this research may be applied in dental clinic daily practice by improving the bonding between the third-generation zirconia prosthesis and the dental tissues to ensure longer durability of the prosthesis.

The current study showed that the surface roughness values ​​in the first group using the Nd:YAG laser were greater than in the continuous wave CO_2_ laser roughening group, with a statistically significant difference.

The current study agreed with Arami et al. that the roughening with the Nd:YAG laser was stronger than the CO_2_ laser and would provide superior bond strength, as the two Nd:YAG laser groups (2.5W-2W) recorded higher surface roughness compared to other groups that used lasers [13]. The study concluded that the greater the laser power, the greater the surface roughness. This may be due to the fact that the use of the Nd:YAG laser has a stronger effect on hard tissues than the effect of the CO_2_ laser, which is used to treat soft tissues [14].

The current study differed from Hemdan et al., which evaluated the effect of different surface parameters of cubic zirconia on surface roughness and bond strength after treating the surface with sandblasting or a CO_2_ laser [15]. The results showed that roughening using a CO_2_ laser had the highest shear strength of resin cement with zirconia ceramics, followed by Sandblasting with aluminum oxide and this differs from the results of the current study. This difference may be attributed to the difference in the laser power used. In the current study, a CO_2_ laser power of 4 watts was used, while in his study, the power used was 10 watts and 20 watts [15].

The current study agreed with the study of Hadi Ran, which studied the effect of roughness of the surface of zirconia with CO_2_ and Nd:YAG lasers and evaluated the surface roughness and bond strength. The results showed that the surface roughness was higher in the Nd:YAG laser group compared to the CO_2_ laser, and this is consistent with the results of the current study [16].

This article has several limitations including not conducting scanning electron microscope sections of the laser-treated surfaces of cubic zirconia to ensure that there are no cracks or fissures within them.

## Conclusions

Surface roughness is one of the most important factors that play an important role in increasing the connection between the surface of the tooth and the applied restoration. Due to the increased interest in zirconia and the improvement of its mechanical and aesthetic properties, studies have increased that work to improve and increase its surface roughness so that it can be used as a veneer in the future. Within the limitations of this study, we conclude that the Nd:YAG laser roughens the surface of zirconia to a greater extent than the continuous wave CO_2_ laser, with a substantially significant difference.

## References

[1] Manziuc M-M, Gasparik C, Negucioiu M (2019). Optical properties of translucent zirconia: a review of the literature. Eurobiotech J.

[2] Kwon SJ, Lawson NC, McLaren EE, Nejat AH, Burgess JO (2018). Comparison of the mechanical properties of translucent zirconia and lithium disilicate. J Prosthet Dent.

[3] Stawarczyk B, Keul C, Eichberger M, Figge D, Edelhoff D, Lümkemann N (2017). Three generations of zirconia: from veneered to monolithic. Quintessence Int.

[4] Carrabba M, Keeling AJ, Aziz A, Vichi A, Fabian Fonzar R, Wood D, Ferrari M (2017). Translucent zirconia in the ceramic scenario for monolithic restorations: a flexural strength and translucency comparison test. J Dent.

[5] Al Hamad KQ, Abu Al-Addous AM, Al-Wahadni AM, Baba NZ, Goodacre BJ (2019). Surface roughness of monolithic and layered zirconia restorations at different stages of finishing and polishing: an in vitro study. J Prosthodont.

[6] Kirmali O, Kustarci A, Kapdan A (2015). Surface roughness and morphologic changes of zirconia: effect of different surface treatment. Niger J Clin Pract.

[7] Hmaidouch R, Müller WD, Lauer HC, Weigl P (2014). Surface roughness of zirconia for full-contour crowns after clinically simulated grinding and polishing. Int J Oral Sci.

[8] Yilbas B (2015). Laser treatment of zirconia surface for improved surface hydrophobicity. J Alloys Compd.

[9] Noda M, Okuda Y, Tsuruki J, Minesaki Y, Takenouchi Y, Ban S (2010). Surface damages of zirconia by Nd:YAG dental laser irradiation. Dent Mater J.

[10] Gautam C, Joyner J, Gautam A, Rao J, Vajtai R (2016). Zirconia based dental ceramics: structure, mechanical properties, biocompatibility and applications. Dalton Trans.

[11] Beuer F, Stimmelmayr M, Gernet W, Edelhoff D, Güth J-F, Naumann M (2010). Prospective study of zirconia-based restorations: 3-year clinical results. Quintessence Int.

[12] Keller U, Hibst R, Geurtsen W (1998). Erbium:YAG laser application in caries therapy. Evaluation of patient perception and acceptance. J Dent.

[13] Arami S, Tabatabae MH, Namdar SF, Chiniforush N (2014). Effects of different lasers and particle abrasion on surface characteristics of zirconia ceramics. J Dent (Tehran).

[14] Rezazadeh F, Dehghanian P, Jafarpour D (2019). Laser effects on the prevention and treatment of dentinal hypersensitivity: a systematic review. J Lasers Med Sci.

[15] Hemdan E, Morsy T, Mohamed F (2021). The effect of different surface treatments on shear bond strength of cubic zirconia. Egypt Dent J.

[16] Ranjzad H, Heidari B, Rad FO, Hendi A, Ghorbani Z (2022). Evaluation of effect of zirconia surface treatment with CO(2) and Nd:YAG lasers on shear bond strength between zirconia frameworks and porcelain veneers. J Contemp Dent Pract.

